# Strengthening timely detection and reporting of unusual respiratory events from health facilities in Yaoundé, Cameroon

**DOI:** 10.1111/irv.12684

**Published:** 2020-01-10

**Authors:** Karen A. Alroy, Luc Christian Gwom, Chanceline Bilounga Ndongo, Sebastien Kenmoe, Gwladys Monamele, Alexey Clara, Brett Whitaker, Henri Manga, Carolle Yanique Tayimetha, Dorine Tseuko, Bienvenu Etogo, Omer Pasi, Alain Georges Etoundi, Elise Seukap, Richard Njouom, Arunmozhi Balajee

**Affiliations:** ^1^ Division of Viral Diseases National Center for Immunization and Respiratory Diseases Centers for Disease Control and Prevention Atlanta GA USA; ^2^ Division for the Fight against Disease, Epidemics and Pandemics Ministry of Health Yaoundé Cameroon; ^3^ Centre Pasteur du Cameroun Yaoundé Cameroon; ^4^ National Public Health Laboratory Ministry of Health Yaoundé Cameroon; ^5^ Division of Global Health Protection Center for Global Health Atlanta GA USA

**Keywords:** cameroon, early warning and response, event‐based surveillance, global health security, health facility, surveillance

## Abstract

**Background:**

The International Health Regulations state that early detection and immediate reporting of unusual health events is important for early warning and response systems.

**Objective:**

To describe a pilot surveillance program established in health facilities in Yaoundé, Cameroon in 2017 which aimed to enable detection and reporting of public health events.

**Methods:**

Cameroon’s Ministry of Health, in partnership with the US Centers for Disease Control and Prevention, Cameroon Pasteur Center, and National Public Health Laboratory, implemented event‐based surveillance (EBS) in nine Yaoundé health facilities. Four signals were defined that could indicate possible public health events, and a reporting, triage, and verification system was established among partner organizations. A pre‐defined laboratory algorithm was defined, and a series of workshops trained health facilities, laboratory, and public health staff for surveillance implementation.

**Results:**

From May 2017 to January 2018, 30 signals were detected, corresponding to 15 unusual respiratory events. All health facilities reported a signal at least once, and more than three‐quarters of health facilities reported ≥2 times. Among specimens tested, the pathogens detected included *Klebsiella pneumoniae*, *Moraxella catarrhalis*, *Streptococcus pneumoniae*, *Haemophilus influenza*, *Staphylococcus aureus*, *Pneumocystis jiroveci*, influenza A (H1N1) virus, rhinovirus, and adenovirus.

**Conclusions:**

The events detected in this pilot were caused by routine respiratory bacteria and viruses, and no novel influenza viruses or other emerging respiratory threats were identified. The surveillance system, however, strengthened relationships and communication linkages between health facilities and public health authorities. Astute clinicians can play a critical role in early detection and EBS is one approach that may enable reporting of emerging outbreaks and public health events.

## 1 INTRODUCTION

In May 2016, an outbreak of avian influenza A (H5N1) virus “H5N1” occurred among poultry in Yaoundé, the capital city of Cameroon. The outbreak caused widespread poultry mortality, required depopulation on select poultry farms, and resulted in the death of >15 000 birds.^1^ The die‐off was detected by Cameroon's livestock services. Cameroon's Ministry of Health (MOH) was rapidly notified and within 24 hours activated their public health emergency operations center for rapid investigation and response for potential infection in humans.^2^ Public health officials closely monitored individuals with poultry exposure and ultimately, no humans with influenza A (H5N1) virus infection were diagnosed.

At the time, Cameroon had two surveillance systems that may have detected severe respiratory disease in humans in the circumstance that avian‐to‐human transmission occurred. The MOH's Division for the Fight against Disease, Epidemics and Pandemics (Direction de la Lutte Contre la Maladie, les Epidémies et les Pandémies [DLMEP]) coordinates a national surveillance platform for integrated disease surveillance and response (IDSR), where cases of priority diseases are counted weekly and registered at the national level.^3^ Each health facility is expected to routinely report aggregate IDSR data and immediately notify DLMEP if a suspected outbreak occurs. The other surveillance system was an indicator‐based sentinel surveillance for influenza, coordinated by the Centre Pasteur du Cameroun (CPC), Cameroon's national influenza center, where 16 hospitals (five of which were in Yaoundé) utilize the World Health Organization (WHO) standard case definitions for severe acute respiratory infection (SARI) and/or influenza‐like illness (ILI) to detect possible influenza cases.^4^, ^5^, ^6^

While the H5N1 outbreak illustrated rapid notification from the animal to human health sectors, the situation raised important questions about detection and reporting of potential human infections with avian influenza from hospitals. The existing IDSR and influenza surveillance systems in theory contributed to routine counting of SARI, ILI, and atypical respiratory cases; however, neither system reliably notified the MOH of suspected outbreaks in a timely way, despite specific instructions to do so within IDSR. During the H5N1 outbreak, it was unclear whether Yaoundé health facilities would recognize potential human infections with avian influenza, and if they did, whether the MOH would be alerted rapidly to initiate control measures. To improve timely detection and immediate notification of suspected outbreaks, the MOH, with support from the United States Centers for Disease Control and Prevention (CDC), implemented an event‐based surveillance (EBS) program focused on detection of unusual respiratory events in Yaoundé.

Event‐based surveillance systems, whether web or media based, focused at the community or healthcare facility level, are characterized by early detection and immediate reporting of potential public health events. They are seldom disease or pathogen‐specific, and instead rely on pattern recognition.^7^ While much of the EBS literature focuses on web or media EBS,^8^, ^9^, ^10^ and community EBS,^8^, ^11^, ^12^, ^13^, ^14^ few reports describe the implementation of EBS in healthcare facilities.^13^, ^14^ The EBS program in Yaoundé was a collaboration among DLMEP, CPC, Cameroon's National Public Health Laboratory (Laboratoire National de Santé Publique de Cameroun, LNSP), and CDC. This report describes the implementation process, signal detection data, and the strengths and challenges of the program.

## 2 METHODS

Within the context of EBS generally, the WHO defines a signal as any data or information that could represent a potential acute risk to human health.^7^ Each reported signal undergoes a process of triage and verification in order to ensure that a true public health event is occurring before public health authorities are activated.^7^ The data sources that contribute to an EBS system (web, media, community, healthcare facility, etc) will influence the design of the data collection process and how the system will be tailored for its intended audience.^7^

In this EBS system focused on healthcare facilities, availability of signal definitions can guide healthcare workers to detect potential health events. Therefore, as a first step of implementing respiratory‐focused EBS, the DLMEP defined four signals that could indicate a potential public health event (Table 1). The signals were focused on (a) healthcare worker illness, (b) illness clusters, (c) zoonotic transmission, and (d) unusual respiratory cases. The DLMEP selected health facilities within Yaoundé to participate in the program, including private, public, military, and religious institutions.

**Table 1:** Signals requiring an immediate report to Centre Pasteur du Cameroun by health facilities in Yaoundé

Signals
Any unexplained severe respiratory illness in a healthcare worker who has been exposed to hospitalized patients with respiratory illness. Any cluster (≥2 people within 1 week) of patients in the same family, social group, or work setting with severe acute‐onset respiratory illness that requires hospitalization. Any patient with recent exposure to sick or dead animals with severe acute‐onset respiratory illness that requires hospitalization. Any unusual cases of severe acute‐onset respiratory illness, including Increases in number of unexplained deaths Increases in intensive care unit admissions for respiratory illness Increases in treatment failure, including unexplained worsening and/or rapidly progressive pneumonia in an individual patient

The primary selection criterion was for health facilities to have the capacity to receive, treat, and hospitalize patients with severe respiratory illness. Cameroon's MOH selected nine health facilities to include in the EBS program (Table 2). Each facility appointed an individual focal point who served as the primary coordinator for their institution. The DLMEP provided these focal points with a hotline to report signals on the same day of detection. Mobile telephones and phone credit were given to the healthcare facility focal points to enable immediate reporting.

**Table 2:** Characteristics of health facilities and summary of signals detected through event‐based surveillance in Yaoundé, Cameroon March 2017–January 2018

Hospital code	Hospital type	Number of signals reported	Number of verified signals	Number of events
Hospital A	Public pediatric hospital	7	7	7
Hospital B	Private hospital	6	6	3
Hospital C	Public university teaching hospital	4	1	0
Hospital D	Public pediatric and obstetric hospital	4	1	1
Hospital E	Public military hospital	3	3	2
Hospital F	Public hospital	2	1	0
Hospital G	Public hospital	2	1	1
Hospital H	Public hospital	1	0	0
Hospital I	Religious hospital	1	1	1
Total		30	21	15

After defining the signals and selecting pilot health facilities, two training courses to operationalize and launch the EBS program were conducted. The first course was a 2‐day national‐level training of trainers hosted by DLMEP with technical support from CDC, and held in Yaoundé, Cameroon in February 2017. Participants were leadership and operational staff from DLMEP, CPC, and LNSP. This course taught fundamental EBS concepts—how to conduct triage and verification when signals are reported—as well as how to teach signal detection and reporting to the health facility staff at the subsequent training.

In March 2017, the second 1‐day course was held in Mfou, Cameroon, outside of the capital city. The participants included health facility focal points and clinicians and were taught by DLMEP, CPC, and LNSP representatives who had participated in the national‐level training of trainers. The second course was focused on signal detection and reporting, and everyone received posters describing the four signals. Participants were instructed to hang the posters at their health facilities and sensitize colleagues in signal detection. The EBS program was launched in May 2017 when health facility focal points received mobile telephones.

**Figure 1:**
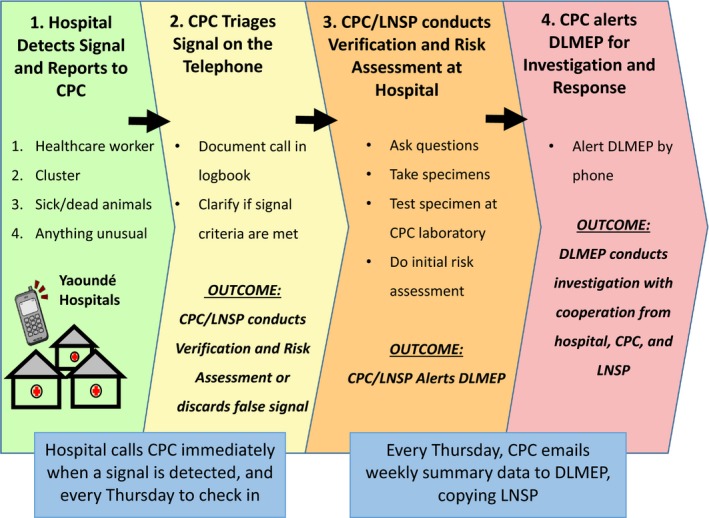
Schematic diagram of information flow and reporting structure in health facility event‐based surveillance in Yaoundé, Cameroon. CPC, Centre Paseur du Cameroun;Pasteur Center of Cameroon; DLMEP: MINSANTE Direction de la Lutte Contre la Maladie, les Epidémies et les Pandémies; Ministry of Health’s Division for the Fight against Disease, Epidemics and Pandemics; LNSP, Laboratoire National de Sante Publique de Cameroun; Cameroon’s National Public Health Laboratory

Within the EBS framework, when a clinician or nurse detected a signal, they were instructed to notify the healthcare facility EBS focal point, who would immediately call CPC to report it (Figure 1). When CPC received the telephone call, they conducted the process of triage while on the phone, confirming that one or more of the four signals were met and that the current signal did not represent a duplicate of a previously reported signal (ie, that it was a true signal). For all true signals, the CPC and LNSP worked cooperatively to conduct the process of verification. This entailed traveling to the healthcare facility to interview the clinician and patient to determine if the situation represented a threat to public health.

During each verification, CPC/LNSP completed a verification form to collect basic information such as the number of human cases, types of symptoms, severity of illness, and exposure history. Laboratory testing is not an essential component of EBS verification; however, the pilot had the capacity to include it. While at the healthcare facility, CPC/LNSP would collect nasopharyngeal and oropharyngeal (NP/OP) specimens. Specimens were taken from all people involved in the signal. Following verification, if CPC/LNSP determined the situation as a public health threat, the signal was categorized as an event.

After specimens were collected, all samples were immediately transported by portable cooler to the Virology Laboratory at CPC and analyzed according to a pre‐defined algorithm. Specimens were first tested for influenza A/B viruses using in‐house real‐time reverse transcriptase polymerase chain reaction (rRT‐PCR) assays. Influenza‐positive specimens underwent additional subtyping. Influenza‐negative specimens were tested with a multiplex platform, the Fast Track Diagnostic 33 (FTD33; Fast Track Diagnostics, Luxembourg), a commercial rRT‐PCR testing kit capable of detecting 21 respiratory viruses and 12 bacteria. Patients with negative test results underwent additional pathogen detection if the clinical circumstances warranted further investigation. To strengthen diagnostic capacity in preparation for EBS, as well as for general public health preparedness, CPC participated in an advanced training on rRT‐PCR diagnostic techniques and the use of multiplex platforms hosted by CDC in July 2016 in Atlanta, Georgia. The FTD kits were provided free of cost through the International Reagent Resource (https://www.internationalreagentresource.org/).

Health facilities were not mandated to maintain a register or documentation of detected signals at their healthcare facility. Their primary responsibility was signal detection and immediate reporting to CPC. Both CPC and LNSP, however, maintained a logbook for tracking signals and events. CPC and LNSP worked in tandem to strengthen ties between the two laboratories, as well as to build connections with health facilities.

Every week, even when no signals were detected, each health facility focal point communicated by telephone with CPC or LNSP for zero reporting. This weekly call was an opportunity to confirm that no signals were detected that week as well as to maintain open lines of communication. CPC then submitted a weekly report on EBS for the DLMEP surveillance meeting each Friday. These data were additionally presented during the weekly Monday coordination meeting led by the top MOH leadership. Five months after implementation, DLMEP conducted site visits at each health facility to discuss EBS with focal points and clinicians. These site visits culminated with a DLMEP‐hosted refresher training workshop in October 2017 in Mfou that brought together all health facility focal points. This workshop offered supportive supervision and allowed for knowledge sharing among the healthcare facility staff. The EBS pilot continued until January 2018.

No ethical approval was required because the data used in this manuscript were from public health surveillance.

## 3 RESULTS

From May 2017 to January 2018, 30 signals were detected and reported (Table 2). All healthcare facilities reported at least one signal, and more than three‐quarters of the facilities reported two or more signals. Signal four, “any unusual cases of severe acute‐onset respiratory illness” was reported 25 times (83.3% of all signals), and it was routinely reported throughout the 9‐month pilot. The signal corresponding to a cluster of patients with respiratory disease was reported three times (10%), and the signal corresponding to healthcare worker illness was reported twice (6.7%). During the triage process, nine of the 30 signals (30%) were determined to be false and 21 (70%) were considered true signals that warranted collection of additional information. The signals that were determined to be false were so categorized because they did not match signal criteria; no duplicate signals were reported.

Following the verification process, 15 of the 21 true signals were verified by CPC and LNSP and reported to DLMEP as events. Of the 15 events, 14 were detected by the signal of an unusual case of acute respiratory illness, and one was detected by the signal of severe respiratory illness in a healthcare worker after exposure to a patient. The patient demographics of the events were as follows: 6 females, 8 males, and 1 person with missing sex, with a median age of 3 years (interquartile range: 3 months to 26 years). Of these events, 11 cases presented with symptoms suggestive of severe pneumonia with additional complications such as malaria, diarrhea, vomiting, convulsions, respiratory distress, or death.

Among the 21 true signals that underwent verification, including the two signals of reported clusters, a total of 23 NP/OP specimens were collected and tested. Fifteen (65.2%) of the 23 specimens tested had a positive laboratory result. Six (26.0%) were positive for influenza A (H1N1) virus. All specimens positive for influenza A (H1N1) virus were collected in October, the start of Cameroon's rainy season. While a single pathogen was detected in four specimens, co‐detection was common: four had two pathogens, five had three pathogens, and two had four pathogens. Among specimens tested, the pathogens identified included *Klebsiella pneumoniae*, *Moraxella catarrhalis*, *Streptococcus pneumoniae*, *Haemophilus influenza*, *Staphylococcus aureus*, *Pneumocystis jiroveci*, influenza A (H1N1) virus, rhinovirus, and adenovirus.

## 4 DISCUSSION

The H5N1 outbreak that occurred among poultry in 2016 resulted in rapid notification from animal to human health sectors, demonstrating strong one health multi‐sectoral collaboration for avian influenza, one of the country's prioritized zoonotic diseases.^15^ Yet, there was concern that rapid reporting from healthcare facility to public health officials of potential respiratory outbreaks in humans was not routinely occurring. Prior to EBS, large Yaoundé health facilities seldom communicated unusual cases to the public health authorities, with poor adherence to IDSR reporting requirements. Cameroon's MOH implemented EBS in Yaoundé to enable rapid detection and notification of emerging respiratory events, and to the best of the authors’ knowledge, this is the first report that focuses on EBS implementation with healthcare facilities providing the primary source of data. Improving capacity for detection of public health events is an important component of the International Health Regulations, a 2005 global agreement to improve health security.

Similar to EBS in other countries and settings, the signals in this system were defined to enable healthcare workers to report when they identify unusual occurrences or patterns.^14^ Astute clinicians can play a critical role in early detection and reporting of emerging outbreaks and public health events.^16^, ^17^ In 2003, a WHO doctor in Vietnam recognized epidemic potential in unusual and severe cases of pneumonia in patients and healthcare workers, and helped to identify the initial spread of severe acute respiratory syndrome (SARS) outside of Hong Kong.^18^ Recently in 2018 in Kerala, India, clinicians quickly recognized a cluster of neurologic cases and rapidly identified the first Nipah virus outbreak in the region.^19^ Empowering physicians to identify potential public health events by relying on their clinical intuition and experience can be facilitated through EBS when healthcare staff have the awareness and mechanisms to communicate these findings to public health authorities.

During pilot implementation, the DLMEP demonstrated that EBS in healthcare facilities can be practical and easy to implement and that health facility staff will engage in rapid detection and notification when provided with simple guidance and a clear reporting mechanism. While the reporting from the clinical to public health sectors can be a challenge worldwide,^20^, ^21^, ^22^ EBS can contribute to improving hospital‐based immediate reporting of health events. The description of EBS implementation presented here can serve as a model for other public health jurisdictions interested in strengthening immediate reporting and notification from health facilities.

There may be a number of reasons as to why an event‐based surveillance approach may be more effective than indicator‐based surveillance systems for rapidly detecting emerging events and/or outbreaks in resource‐limited health facility settings. First, EBS does not require clinicians to follow a specific case definition, but rather encourages medical professionals to draw upon their clinical training and experience to recognize that something is unusual or unexpected. The signals in EBS are designed for clinicians to recognize patterns and unusual trends, in contrast to traditional surveillance case definitions, which are based on disease‐specific identification (Table 1). Second, by utilizing a short list of signals and not requiring any documentation at the health facility, it was easy for clinical staff to engage with the EBS system. The health facility was simply required to call the public health authorities when they detected one or more of the signals. Lastly, health facility EBS systems do not require laboratory confirmation as a condition of reporting. While this pilot integrated a mechanism for laboratory testing during the verification process, outside of the capital, Cameroon does not have reliable laboratory capacity, and surveillance systems that rely on laboratory confirmation could potentially delay reporting from healthcare facilities. By incorporating laboratory testing, however, as a component of verification, clinicians in this pilot gained a non‐financial incentive to participate in EBS, that is, access to laboratory testing for their patients that might not have otherwise been available.

The events detected in this pilot were caused by routine respiratory bacteria and viruses, and no novel influenza viruses or other emerging respiratory threats were identified. DLMEP did not consider any of the events to warrant additional public health action. Despite this, however, an important outcome of the pilot was that EBS helped promote behavior change among clinicians and health facility staff toward detection and reporting of potential outbreaks. An example of this was at health facility E, where a suspected hospital‐acquired infection was recognized and reported as a potential public health threat within 24 hours of detection. The EBS system enabled the clinician to recognize that a potential hospital‐acquired infection could be a risk to public health and that notification was warranted and provided a mechanism to report to public health officials. While the doctor was ultimately diagnosed with rhinovirus, and the situation was deemed low risk, the clinician's heightened public health awareness helped to strengthen communication and reporting practices.

Half of the signals detected, 15 out of 30 (50%), were determined to be events by CPC/LNSP and were reported to DLMEP. This finding is consistent with EBS systems where triage and verification take place at healthcare facilities, such as in Ethiopia where 64% of EBS reports from health centers were verified and found to be true events.^13^ In Ratnayake et al, where community EBS was implemented for detection of Ebola virus disease, approximately 2% of signal alerts corresponded to a patient who met the case definition of suspected, probable or confirmed case.^11^ In our study, a large proportion of the reported signals were determined to be events, however, because clinicians and health facility staff are trained in health and medicine, their judgment of what constitutes a signal and when to report may represent an early type of filtering, reducing background noise.

The implementation of EBS in health facility, however, was not without challenges. The pilot had an initial investment cost, including the cost of trainings, posters, phones, and phone credits as well as in‐kind contribution of laboratory reagents from CDC. Additionally, after the pilot launch, signals were routinely detected and reported by the health facilities for the first 5 weeks. There was a drop in reporting after the initial weeks, which improved again following a DLMEP refresher training. A pattern of waning clinician engagement was observed, likely because of competing clinical demands and responsibilities. This illustrated that the relationship between health facilities and the public health system can be delicate, but that supportive supervision and feedback from the MOH can help to improve motivation. A reliance on supportive supervision for improved performance is noted in other surveillance systems.^23^, ^24^ Fostering hospital engagement and relationships contributes to continued reporting and program sustainability.

Another challenge experienced in EBS implementation was one of physical infrastructure. Telephone landlines are not commonplace in Cameroon, therefore the health facility staff lacked a reliable and free mechanism for reporting. Since 2010, the WHO has instituted a system of float telephones throughout the public health structure in Cameroon that are free of charge to users^25^; however, these were not always available in pilot health facilities for the purpose of EBS. While this EBS pilot program provided mobile telephones and phone credit, this may be problematic on a larger scale since the MOH is not able to equip all health facilities within the country with a cellular telephone and phone credit.

Lastly, during the pilot, both CPC and LNSP provided an important service in conducting triage and verification of reported signals. This role is typically the responsibility of public health epidemiologists, not laboratorians. However, in the context of this pilot and with limited public health human resources at DLMEP, both of these laboratories provided their services. Ultimately, EBS is most sustainable when it can be integrated into existing public health communication structures.

Based on the experience gained from this pilot, DLMEP is scaling up EBS in health facilities in multiple regions throughout the country and plans to formally integrate EBS reporting into Cameroon's IDSR platform. Event‐based surveillance is an essential capacity for a country to rapidly identify and appropriately respond to public health events. Event‐based surveillance systems can be tailored to the specific needs of a country: designed broadly to detect all hazards, or with a particular focus, such as respiratory diseases in this pilot surveillance program.

## 5 DISCLAIMER

The findings and conclusions in this report are those of the authors and do not necessarily represent the official position of the Centers for Disease Control and Prevention.
